# Single-Cell Responses to Face Adaptation in the Human Medial Temporal Lobe

**DOI:** 10.1016/j.neuron.2014.09.006

**Published:** 2014-10-22

**Authors:** Rodrigo Quian Quiroga, Alexander Kraskov, Florian Mormann, Itzhak Fried, Christof Koch

**Affiliations:** 1 Centre for Systems Neuroscience, University of Leicester, 9 Salisbury Rd, Leicester, LE1 7QR, UK; 2 Intitute of Neurology, University College London, Queen Square, London, WC1N 3BG, UK; 3 Department of Epileptology, University of Bonn, Sigmund-Freud-Str. 25, 53105 Bonn, Germany; 4 Department of Neurosurgery and Semel Institute for Neuroscience and Human Behavior, University of California Los Angeles, 760 Westwood Plaza, Los Angeles, CA 90024, USA; 5 Functional Neurosurgery Unit, Tel-Aviv Medical Center and Sackler Faculty of Medicine, Tel-Aviv University. 6 Weizmann Street, 64239 Tel Aviv, Israel; 6 Division of Biology, California Institute of Technology, Pasadena, CA 91125, USA; 7 Allen Institute for Brain Science, Seattle, WA 98103, USA

## Abstract

We used a face adaptation paradigm to bias the perception of ambiguous images of faces and study how single neurons in the human medial temporal lobe (MTL) respond to the same images eliciting different percepts. The ambiguous images were morphs between the faces of two familiar individuals, chosen because at least one MTL neuron responded selectively to one but not to the other face. We found that the firing of MTL neurons closely followed the subjects’ perceptual decisions—i.e., recognizing one person or the other. In most cases, the response to the ambiguous images was similar to the one obtained when showing the pictures without morphing. Altogether, these results show that many neurons in the medial temporal lobe signal the subjects’ perceptual decisions rather than the visual features of the stimulus.

## Highlights

• We used a face adaptation paradigm to bias the perception of ambiguous images
• Neurons in the human MTL fired according to the subjects’ perceptual decisions
• In most cases, ambiguous images triggered similar responses as the original pictures
• MTL neurons follow subjective perception rather than visual inputs

## Teaser

Using an adaptation paradigm to bias the perception of morphed faces, Quian Quiroga et al. show how single neurons in the human medial temporal lobe follow the subjective perception by the subjects rather than the visual features of the stimulus.

## Introduction

A key function of the brain is to extract meaning from relatively limited, noisy, and ambiguous sensory information. We indeed perceive—and are aware of seeing—the face of a particular person rather than the combination of pixels and specific features that compose the person’s face. This process of extracting meaning involves categorizations and perceptual decisions (Beale and Keil, 1995, Freedman et al., 2001, Freedman et al., 2002, Fabre-Thorpe, 2003, Palmeri and Gauthier, 2004, Rotshtein et al., 2005, Heekeren et al., 2008), where similar visual inputs, like the front view of two different faces, can lead to different percepts and, conversely, disparate images, like the front and profile view of a person, give the same percept. Converging evidence has demonstrated the involvement of the ventral visual pathway—going from primary visual cortex to inferotemporal cortex—in visual perception (Logothetis and Sheinberg, 1996, Tanaka, 1996, Tsao and Livingstone, 2008). At the top of the hierarchy along the ventral visual pathway, high-level visual areas have strong connections to the medial temporal lobe (MTL) (Saleem and Tanaka, 1996, Suzuki, 1996, Lavenex and Amaral, 2000), which has been consistently shown to be involved in semantic memory (Squire and Zola-Morgan, 1991, Nadel and Moscovitch, 1997, Squire et al., 2004). It is precisely in this area where we previously reported the presence of “concept cells”—i.e., neurons with highly selective and invariant responses that represent the meaning of the stimulus. In fact, concept cells are selectively activated by different pictures of a particular person, by the person’s written or spoken name, and even by internal recall, in the absence of any external stimulus (Quian Quiroga et al., 2005, Quian Quiroga et al., 2008a, Quian Quiroga et al., 2009, Gelbard-Sagiv et al., 2008, Quian Quiroga, 2012).

In the quest to understand how the brain constructs meaning from sensory information, several works have studied the firing of single neurons in monkeys using identical but ambiguous stimuli that elicit different perceptual outcomes (for reviews, see Logothetis, 1998, Kanwisher, 2001, Blake and Logothetis, 2002). One such experimental manipulation is the use of face adaptation, where the perception of an ambiguous face is biased by the presentation of another face shortly preceding it (Leopold et al., 2001, Leopold et al., 2005, Webster et al., 2004, Moradi et al., 2005, Jiang et al., 2006, Fox and Barton, 2007, Webster and MacLeod, 2011). In this work, we used the unique opportunity of recording the activity of multiple single neurons in awake human subjects—who were implanted with intracranial electrodes for clinical reasons—to study how neurons in the MTL respond to face adaptation. In particular, starting from two pictures of persons known to the subject (for which we had a neuron firing to one of them but not to the other), we created ambiguous morphed images that were briefly flashed, immediately following the presentation of an adaptor image (one of the two pictures). Given the high-level representation by cells in this area, we asked whether, and to what extent, the firing of MTL neurons follows the perceptual decision by the subjects.

## Results

**Figure 1:**
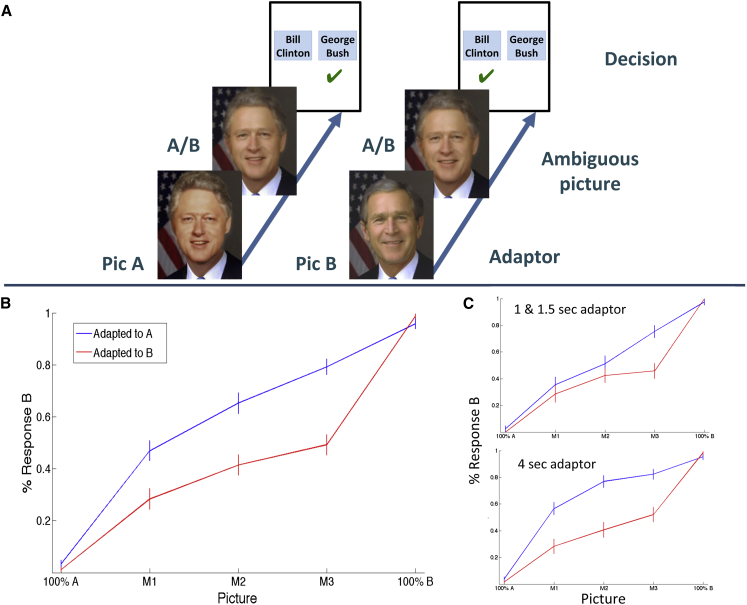
Behavioral Results (A) Adaptation paradigm. The perception of an ambiguous morphed image (A/B) was biased by the previous presentation of one of the pictures used to generate the morphing (picture A or picture B). The task of the subjects was to respond whether they recognized the ambiguous picture as A or B (here, presidents Bill Clinton and George Bush). (B) Mean percentage of trials in which subjects recognized the ambiguous image as B, when previously adapted to picture A (blue) or B (red). For comparison, the responses to the nonambiguous picture presentations (100% A and 100% B, likewise preceded by the adaptors) are also shown. (C) Same as (B) but separating between the 1–1.5 s and the 4 s presentation of the adaptors. The longer presentation of the adaptors led to a larger perceptual bias, namely the tendency to recognize the morphed picture as B when adapted to A (and vice versa). Error bars denote SEM.

Subjects saw the presentation of ambiguous morphed images (e.g., a morph between presidents Bill Clinton and George Bush) preceded by an adaptor (the picture of Clinton or the one of Bush) and had to respond whether the ambiguous picture corresponded to one or the other (Figure 1A). Figure 1B shows the overall behavioral responses obtained in 21 experimental sessions with ten subjects for the three degrees of morphing used. In agreement with previous work (Leopold et al., 2005), subjects tended to identify the ambiguous morphed pictures (M1, M2, and M3) as the opposite of the adaptor. That means, for each morphing, the adaptation to picture A led to a significantly higher recognition of the ambiguous picture as B (and vice versa) (M1: p < 10^−3^; M2: p < 10^−4^; M3: p < 10^−7^; Wilcoxon rank-sum test). This perceptual difference was larger for longer presentations of the adaptors (Figure 1C).

Given the different perceptual outcomes using the same set of ambiguous images, we then asked whether the firing of single neurons in the medial temporal lobe was entirely driven by visual features or whether it was modulated by the subjects’ decision (picture A or B). Altogether, we obtained 81 significant responses (defined as a statistical significant response to a specific face; see Experimental Procedures) in 62 units (45 units with 1 response, 15 with 2, and 2 units with 3 responses): 26 in the hippocampus, 20 in the entorhinal cortex, 15 in the parahippocampal cortex, and 20 in the amygdala.

**Figure 2:**
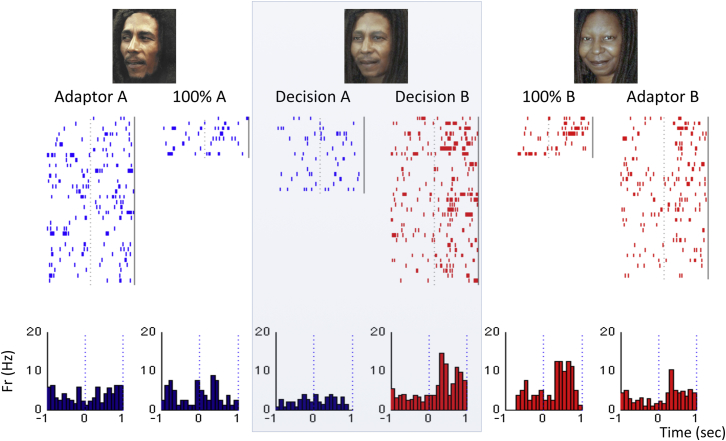
Single Neuron Exemplary Responses Responses of a single unit in the hippocampus that fired strongly to the presentations of the picture of Whoopi Goldberg (100% B) but not to Bob Marley (100% A). The responses of the neuron to the pictures when used as adaptors (Adaptor A, Adaptor B) are also displayed. The unit had a larger response to the ambiguous pictures (M1, M2, and M3 pulled together; middle plots) when the subject recognized them as Goldberg (Decision B) compared to when he recognized them as Marley (Decision A). Based on the single-trial firing upon the presentation of the ambiguous pictures, a linear classifier could predict the subject’s decision significantly better than chance (p < 10^−3^; see Experimental Procedures). (See also Figures S1, S2, and S3 for additional examples.)

Figure 2 shows the responses of a single unit in the hippocampus during the adaptation paradigm. The neuron fired selectively to actress Whoopi Goldberg (picture B) when shown without morphing (100% B; mean: 7.37 spikes/s) and did not respond to Bob Marley (100% A; mean: 3.87 spikes/s). The middle columns (highlighted) show the responses to the morphed pictures separated according to the subject’s response (recognized A or B). Even though the ambiguous pictures were exactly the same, there was a larger activation of the neuron when the subject reported recognizing them as Goldberg (mean: 7.84 spikes/s) compared to when he recognized them as Marley (mean: 2.40 spikes/s). In line with this observation, a linear classifier could correctly predict the subject’s response upon the presentation of the ambiguous morphed pictures in 77% of the trials, which is significantly better than chance with p < 10^−3^ (see Experimental Procedures). We applied the linear classifier to the 75 out of 81 responses for which we had at least five trials for each decision (recognized A and recognized B). Altogether, the decoding performance was significantly larger than chance with p < 0.05 (see Experimental Procedures) for 23 of the 75 responses (31%).

**Figure 3:**
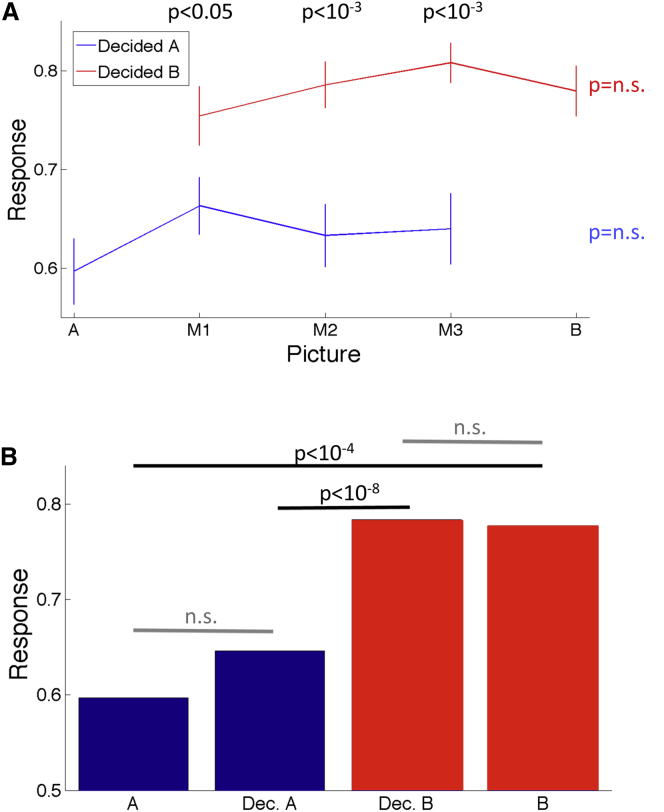
Population Results (A) Mean grand average responses for the three morphs used (M1, M2, and M3) and for the original (nonmorphed) images (A was the one image of the pair that was nonresponsive; while B was the responsive one). For each morph, note the significantly higher responses when the subject reported recognizing the image as B (p values for the average differences were obtained with Wilcoxon rank-sum tests). Error bars denote SEM. (B) Mean response strength for picture A, picture B, and the morphed pictures, separated according to the subjects’ report (Decided A or B). Note again the much higher response strength for the ambiguous pictures when recognized as B, which was similar to the response obtained when showing the original (nonmorphed) picture B. Likewise, the presentation of picture A gave a response that was statistically the same as the one obtained when showing picture A.

A pattern similar to the one in Figure 2 (additional examples are shown in Figures S1, S2, and S3 in the Supplemental Information available online) was found in the average normalized responses (Figure 3). For the three morphed images, M1, M2, and M3, there was a significantly higher activation when the subjects recognized the ambiguous images as person B (responsive) compared to A (nonresponsive) (Figure 3A). Moreover, the response to the three morphed images perceived as picture B did not differ statistically from the one obtained in response to the presentation of picture B without morphing. Similarly, the presentation of picture A (without morphing) elicited a response that did not differ statistically from the one elicited by the morphed images when recognized as A. Figure 3B shows the results pooled together the three morphs used. As before, there was a significantly larger response to picture B and the ambiguous pictures recognized as B, compared to picture A and the ambiguous pictures recognized as A. For each response (A or B) there were no significant differences in the neurons’ firing to the ambiguous and the original (nonmorphed) pictures. These results were consistent across MTL areas. That means, when considering the neurons of each area separately (hippocampus, amygdala, entorhinal cortex, and parahippocampal cortex), in all cases the response to the ambiguous pictures recognized as picture B were significantly larger than when recognized as A, and there were no significant differences in the responses to the original (nonmorphed) pictures A or B and the ambiguous pictures recognized as picture A or B, respectively. This lack of significant differences between the ambiguous and the original pictures should, however, be interpreted with caution, given that such null result could be due to an insufficient number of trials or a large variability in the responses across different neurons, among other factors. To further study this issue, we used a linear classifier to predict the presentation of the original or the ambiguous pictures leading to the same perceptual outcome (recognized A or recognized B). As before, we considered those responses for which we had at least five trials in each condition. In 10 out of 52 cases (19%) the linear classifier could discriminate better than chance (p < 0.05) the presentation of the original picture B from the ambiguous picture recognized as B, whereas in 15 out of 62 cases (24%) the classifier could significantly distinguish between picture A and the ambiguous picture recognized as A.

**Figure 4:**
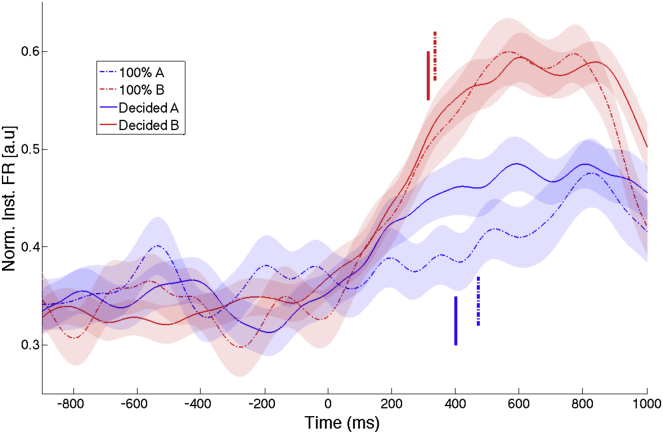
Average Instantaneous Firing Rates Grand average time courses of instantaneous firing rates for each condition: presentation of picture A (100% A), B (100% B), and ambiguous pictures recognized as A and B. Note the similar response pattern for picture B (responsive picture) and the ambiguous picture recognized as B. These responses were higher than the ones to the presentation of A (nonresponsive picture) or the ambiguous picture recognized as A. There were no significant differences in the latency of responses obtained in each condition (vertical lines). Shaded areas around mean values represent SEM.

Complementing these results, in Figure 4 we show the time course of the normalized average instantaneous firing rate curves (see Experimental Procedures) for the four conditions (pictures A or B, and ambiguous pictures recognized as A or B). Note the similarity of the firing rate curves in response to the pure picture B and to the ambiguous pictures recognized as B (difference nonsignificant; Kolmogorov-Smirnov test). These responses were significantly larger than the ones to picture A and to the ambiguous pictures recognized as A (K-S test; p < 10^−10^ in all cases). Note, however, that in this case there is also a lower response to picture A compared to the ambiguous picture recognized as A, which was statistically significant (K-S test; p < 10^−8^). Given these results, it seems plausible to argue that the lack of statistical significance when analyzing the whole response strength (Figure 3B) was due to variability in the different responses, an interpretation that is in line with the cell-by-cell decoding results described in the previous paragraph. The mean response latencies (see Experimental Procedures) for picture B and the ambiguous pictures recognized as B (335 ms and 312 ms, respectively) were not significantly different. The response latencies for picture A and the ambiguous pictures recognized as A were slightly larger (469 ms and 399 ms, respectively) but also not statistically different from each other, or from the responses to picture B.

Finally, to disentangle whether the differential responses to the morphed pictures (according to the subjects’ perception) could, at least in part, be explained by a modulation in the firing of the neurons given by the presentation of the preceding adaptors, we performed a two-way ANOVA with “decision” (recognized A or B) and “adaptor” (picture A or B) as independent factors. This analysis showed that the differential firing of MTL neurons was due to the decision and not due to the preceding adaptor. In fact, there was a significant effect for the factor “decision” (p < 10^−4^) but not for “adaptor” or for the interaction between both factors.

## Discussion

Previous works used face adaptation paradigms (Leopold et al., 2001, Leopold et al., 2005, Webster et al., 2004, Moradi et al., 2005, Jiang et al., 2006, Fox and Barton, 2007, Webster and MacLeod, 2011) or morphing between pictures (Beale and Keil, 1995, Leopold et al., 2001, Leopold et al., 2006, Rotshtein et al., 2005) to study different aspects of visual perception, more specifically, the perception of faces. Faces are indeed particularly relevant for primates, and single-cell recordings in monkeys (Bruce et al., 1981, Perrett et al., 1982, Desimone et al., 1984, Logothetis and Sheinberg, 1996, Tanaka, 1996, Tsao et al., 2006, Tsao and Livingstone, 2008, Freiwald and Tsao, 2010), as well as imaging studies in humans (Kanwisher et al., 1997), have identified specific areas involved in the recognition of faces. We here used face adaptation to bias the perception of ambiguous morphed images to investigate whether such perceptual bias affected the firing of MTL neurons. We indeed found a strong modulation of the responses of these neurons when the subject perceived one person or the other, in spite of the fact that the ambiguous images were exactly the same. In particular, the responses to the ambiguous images were significantly larger when the subject recognized the image as person B (the one for which the neuron originally fired) compared to person A. Interestingly, the responses to the ambiguous images identified as picture B (the one eliciting responses) were not significantly different, both in terms of magnitude and latency, from the ones obtained when showing picture B without morphing. The responses to picture A (the one not eliciting responses) were also not significantly different, in terms of magnitude and latency, from the ones to the ambiguous pictures recognized as A when considering the whole response strength (Figure 3). However, in this case there was a tendency for higher responses to the ambiguous pictures that did reach statistical significance when considering the time-resolved average responses (Figure 4). Thus, the lack of statistical difference with the whole response strength may be attributed to variability in the neurons’ responses. In fact, in about 20% of the cases, a linear classifier could distinguish above chance the presentation of the original and ambiguous pictures leading to the same perceptual decision. Such differences could, in principle, be attributed to a higher cognitive load when deciding the identity of a morphed compared to a nonmorphed image, which could have involved different degrees of attention. It is also possible that, even if eventually making a single decision in each trial, subjects may have had (at least in some cases) an alternating percept between both identities when seeing the morphed pictures. We also observed a smaller difference between the responses to the original and the morphed presentations for the images eliciting responses (picture B) compared to the difference for the images not eliciting responses (picture A), which could in principle be attributed to a firing rate saturation—i.e., there was little modulation in the responses to picture B because the neurons were already close to their maximum firing rates.

Ambiguous percepts have a long history of being used to dissociate neural responses underlying the subjective perception by the subject from the sensory representation of the visual stimuli (Logothetis and Schall, 1989, Leopold and Logothetis, 1996, Logothetis et al., 1996, Logothetis, 1998, Kanwisher, 2001). In this respect, a classic paradigm is binocular rivalry, where two distinct images presented at each eye compete with each other and give rise to a fluctuating perception of one or the other. Single-cell recordings along the ventral visual pathway in monkeys have shown an increase in the number of neurons following the subjective perception in higher visual areas (Logothetis, 1998). Higher visual areas project to the MTL (Saleem and Tanaka, 1996, Suzuki, 1996, Lavenex and Amaral, 2000), where modulations of the neurons’ firing with subjective perception were also found using a binocular rivalry paradigm (Kreiman et al., 2002), and where we previously showed, using short presentation times together with backward masking, that human MTL neurons fired only when the stimulus was recognized and remained at baseline firing levels when it was not (Quian Quiroga et al., 2008b). In this later study, the variability in recognition could be attributed to internal processes, independent of the actual stimulus presentation, and varying degrees of attention. Along this line, we here presented further evidence supporting the claim that MTL neurons follow the subjective perception by the subjects, but in this case using ambiguous images representing competing stimuli—i.e., a morphed image that can be recognized as one person or the other—and modifying the actual perception by means of adaptation.

## Experimental Procedures

### Subjects and Recordings

The data come from 21 sessions in 10 patients with pharmacologically intractable epilepsy. Extensive noninvasive monitoring did not yield concordant data corresponding to a single resectable epileptic focus. Therefore, the patients were implanted with chronic depth electrodes for 7–10 days to determine the seizure focus for possible surgical resection (Fried et al., 1997). Here we report data from sites in the hippocampus, amygdala, entorhinal cortex, and parahippocampal cortex. All studies conformed to the guidelines of the Medical Institutional Review Board at UCLA and the Institutional Review Board at Caltech. The electrode locations were based exclusively on clinical criteria and were verified by CT coregistered to preoperative MRI. Each electrode probe had a total of nine microwires at its end, eight active recording channels, and one reference. The differential signal from the microwires was amplified using a 64-channel Neuralynx system, filtered between 1 and 9,000 Hz and sampled at 28 kHz. Each recording session lasted about 30 min.

### Experimental Paradigm

Subjects sat in bed, facing a laptop computer on which images were presented. The stimuli used were chosen from previous “screening sessions” in which a set of about 100 different pictures of people well known to the subjects (along with several pictures of landmarks, objects, and animals) were shown for 1 s, six times each in pseudorandom order (Quian Quiroga et al., 2005, Quian Quiroga et al., 2007). The pictures used in the screening sessions were partially chosen according to the subject’s interests and preferences. After a fast offline analysis of the data, it was determined which of the presented pictures elicited responses in at least one unit. Between 2 and 5 (mean: 3.14; SD: 0.65) pictures of individuals eliciting responses in the screening sessions were used in the adaptation paradigm reported here. To design the adaptation paradigm, tuned for each patient based on the obtained responses for the selected individuals (e.g., Bill Clinton, Jennifer Lopez), we chose a second person (e.g., George Bush, Jennifer Aniston) and for each stimulus pair (e.g., Bill Clinton and George Bush, Jennifer Lopez and Jennifer Aniston) we created 120 morphed images, going gradually from 100% picture A (Bill Clinton) to 100% picture B (George Bush). Then, we showed the patients each sequence of morphs as a continuous movie (with a presentation rate of 30 frames per s and 4 s per movie) 16 times for each stimulus pair, in pseudorandom order and in both directions, namely, going from picture A to picture B and vice versa. Subjects had to press and hold the down-arrow key to begin the movie presentation, which started 100 ms after the key press, and were instructed to release the key at the moment they recognized the second person. Movies involving the same stimulus pair in either direction were never shown in consecutive trials. Finally, according to the subjects’ responses, for each pair we selected three morphed images (M1, M2, and M3) giving an ambiguous perception: M2 was the one estimated to give the most ambiguous perception to the subject—i.e., the image that corresponded to the mean response time, averaging the presentations going from A to B with the ones going from B to A; M1 and M3 were closer to pictures A and B, respectively, and were between three to eight frames away from M2 (the exact number of frames was heuristically selected in each case to give an ambiguous image but with a slight bias toward recognition of one or the other image). The morphed pictures were created using the software *SmartMorph*, after rescaling and cropping the images with Photoshop. Images were presented at the center of the laptop screen and covered about 1.5° of visual angle.

After determining the morphs eliciting an ambiguous perception, subjects performed the adaptation paradigm, in which the perception of the ambiguous images was biased by first showing one of the two original pictures used to generate the morphs (Figure 1A). The basic idea is that, when shown a morphing between pictures A and B, subjects are more likely to recognize it as picture B if the morphed image is preceded by a presentation of picture A (the adaptor) and vice versa (Webster et al., 2004, Leopold et al., 2005). This effect has been attributed to diminished responses of feature-selective neurons after previous stimulation by the adaptor (Leopold et al., 2005). In the first eight sessions, the adaptor image (either picture A or B of each pair) was shown for 1 and 1.5 s (first six and following two sessions, respectively), but a better perceptual bias was later obtained when using a longer presentation (4 s) of the adaptors (Figure 1C), which was used in the remaining 13 sessions. For each picture pair, a total of six to eight presentations of each morph (M1, M2, and M3) preceded by an adaptation to picture A, and an equal number of times preceded by an adaptation to B, were shown in pseudorandom order. Each trial started with a fixation cross displayed at the center of the screen for 500 ms. After a random delay between 0 and 100 ms, the adaptor picture was presented (for 1, 1.5, or 4 s) and, following a blank of 300 ms, one of the morphed images was shown for 200 ms. In order to compare the responses elicited by the morphed pictures to those to the nonambiguous images, in 15 of the 21 sessions we also added a presentation of the original pictures A and B after the adaptors. The “target images” (M1, M2, M3, 100% A, and 100% B) were followed by a 500 ms blank, after which the names of the two persons of the corresponding stimulus pair were shown and the subject had to indicate which one (s)he perceived with the left/right arrow key (Figure 1A).

### Data Analysis

From the continuous wide-band data, spike detection and sorting were carried out using “Wave_Clus,” an adaptive and stochastic clustering algorithm (Quian Quiroga et al., 2004). As in previous works (Quian Quiroga et al., 2009), a response was considered significant if, for the presentation of the “target images”—either for the 100% A, 100% B (when available), the “recognized A” or “recognized B” presentations (pulling together the responses for the three morphs)—it fulfilled the following criteria: (1) the firing in the response period (defined as the 1 s interval following the stimulus onset) was significantly larger than in the baseline period (the 1 s preceding stimulus onset) according to a paired t test with p < 0.01; (2) the median number of spikes in the response period was at least 2; (3) the response contained at least five trials (given that the number of trials in the conditions “recognized A” and “recognized B” was variable). For the average population plots (Figure 3), we normalized each response by the maximum response across conditions (100% A, 100% B, M1, M2, M3, separated according to the decision: A or B). Statistical comparisons were performed using nonparametric Wilcoxon rank-sum tests (Zar, 1996).

A linear classifier was used to decode the subjects’ decision upon the presentation of the ambiguous morphed images (recognized picture A or B) in those cases where we had at least five trials for each decision. One at a time, the firing in each trial was used to test the classifier, which was trained with the remaining trials (all-but-one cross-validation). As in previous works (Quian Quiroga et al., 2007, Quian Quiroga and Panzeri, 2009), to evaluate the statistical significance of decoding performance, we used the fact that since the outcomes of the predictions of each decision are independent trials with two possible outcomes, success or failure, the probability of successes in a sequence of trials follows the Binomial distribution. Given a probability *p* of getting a hit by chance (p = *1/K*, *K*: number of possible decisions), the probability of getting *k* hits by chance in *n* trials is $P\left(k\right)=\left(\begin{array}{c} n\\ k \end{array}\right)p^{k}{\left(1-p\right)}^{n-k}$, where $\left(\begin{array}{c} n\\ k \end{array}\right)=\frac{n!}{\left(n-k\right)!k!}$ is the number of possible ways of having *k* hits in *n* trials. From this, we assessed statistical significance and calculated a p value by adding up the probabilities of getting *k* or more hits by chance: $\text{p}\text{-}\text{value}=\sum_{j=k}^{n}P\left(j\right)$. We considered a significance level of p = 0.05, thus expecting 5% of the responses to reach significance just by chance. The same procedure was also used to compare the presentation of the original (nonmorphed) and morphed pictures for each perceptual decision (i.e., recognized A versus 100% A, and recognized B versus 100% B).

Instantaneous firing rate curves were calculated by convolving the normalized spike trains with a Gaussian window of 100 ms width. For each response, we estimated the latency onset as the point where the instantaneous firing rate crossed the mean + 2.5 SD of the baseline for at least 100 ms. Similar results were obtained using a threshold of 3 or 4 SD. Statistical differences between the different average firing rate curves were assessed with a Kolmogorov-Smirnov test in the time window from 0 to 1 s after stimulus onset.

## Author Contributions

C.K., I.F., A.K., and R.Q.Q. designed the paradigm; I.F. performed the surgeries; A.K. and F.M. collected the electrophysiological data; R.Q.Q. analyzed the data and wrote the paper; and all authors discussed the results and commented on the manuscript. R.Q.Q. and A.K. contributed equally to the study.

## Supplemental Information

Document S1. Figures S1–S3

Document S2. Article plus Supplemental Information
