# Supplementary material for: Single-Cell Responses to Face Adaptation in the Human Medial Temporal Lobe
Source: Neuron. 2014 Oct 22;84(2):363–9. doi: 10.1016/j.neuron.2014.09.006 (PMC4210637; doi:10.1016/j.neuron.2014.09.006)
Supplement: Document S1. Figures S1–S3 [file mmc1.pdf]

Neuron, Volume *84*

Supplemental Information

Single-Cell Responses to Face Adaptation in the Human Medial Temporal Lobe

Rodrigo Quian Quiroga, Alexander Kraskov, Florian Mormann, Itzhak Fried, and Christof Koch

Supplemental Information

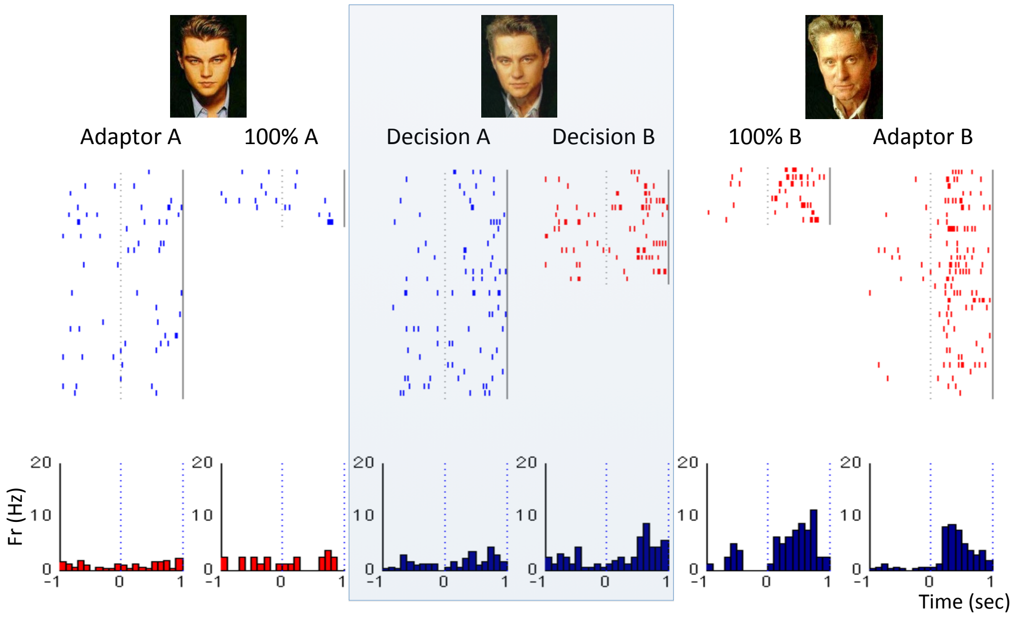

Figure S1, related to Figure 2: A multi unit in the amygdala with a strong activation in response to the picture of Michael Douglas when shown without morphing (100% B, mean: 5.87 spikes/s) or as adaptor (mean: 4.3 spikes/s), and not in response to the picture of Leonardo Di Caprio when shown without morphing (100% A; mean: 1.3 spikes/s) or as adaptor (mean: 1.1 spikes/s). Conventions are the same as for Figure 2. The response to the morphed pictures was larger when the subject recognized them as Douglas (mean: 3.8 spikes/s) compared to when he recognized them as Di Caprio (mean: 1.96 spikes/s). The single trial classifier could predict the subject's responses in 66% of the trials, which is significantly larger than chance ($p<0.05$; see Experimental Procedures).

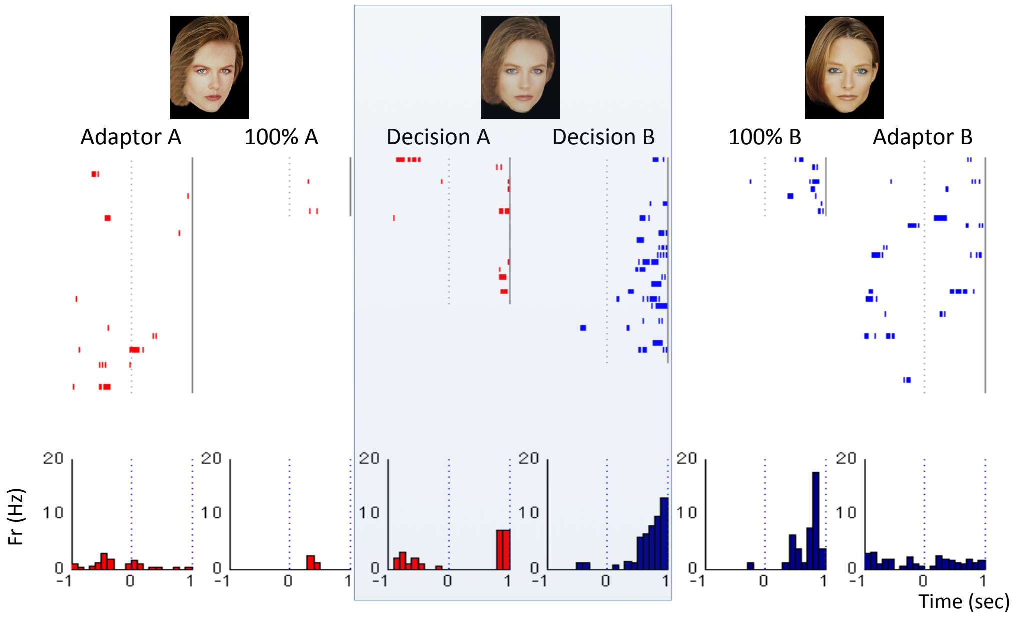

Figure S2, related to Figure 2: A single unit in the hippocampus that showed a strong activation in response to the picture of Jodie Foster when shown without morphing (100% B; mean: 4.1 spikes/s), but with a weaker activation when shown as adaptor (mean: 1.34 spikes/s) or in response to the picture of Nicole Kidman when shown without morphing (mean: 0.37 spikes/s) or as an adaptor (mean: 0.37 spikes/s). Conventions are the same as for Figure 2. The response to the morphed pictures was larger when the subject recognized them as Foster (mean 4.5 spikes/s) compared to when he recognized them as Kidman (mean: 1.4 spikes/s). The single trial classifier could predict the subject's responses in 69% of the trials, which is significantly larger than chance ($p<0.01$).

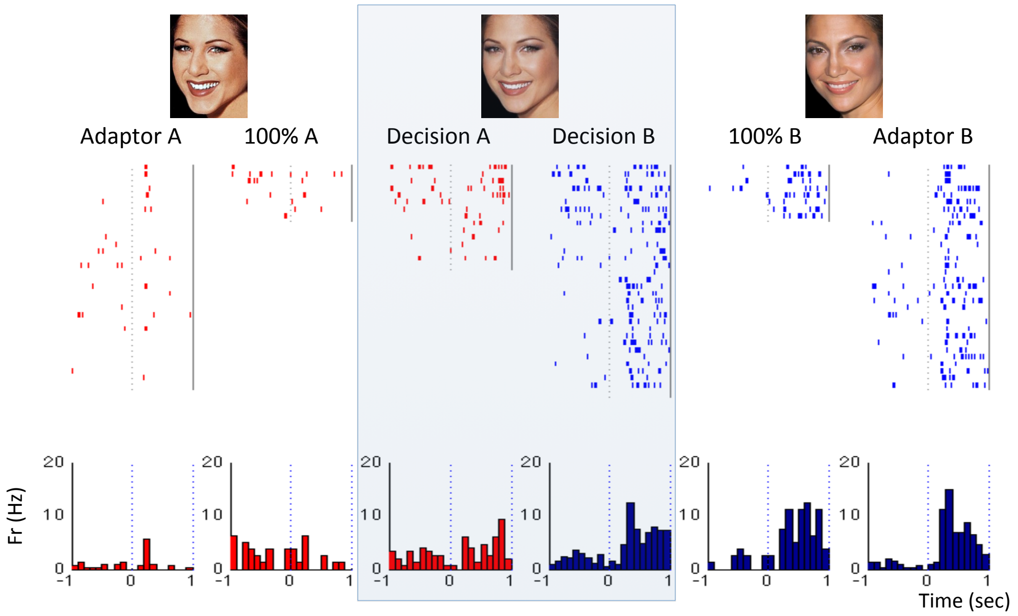

Figure S3, related to Figure 2: A single unit in the amygdala with a strong activation in response to the picture of Jennifer Lopez, both when shown without morphing (100% B; mean: 6.70 spikes/s) or as adaptor (mean: 6.50 spikes/s), and not in response to the picture of Jennifer Aniston when shown without morphing (mean: 1.62 spikes/s) or as an adaptor (mean: 0.80 spikes/s). Conventions are the same as for Figure 2. The response to the morphed pictures was larger when the subject recognized them as Lopez (mean 6.12 spikes/s) compared to when he recognized them as Aniston (mean: 3.70 spikes/s). The single trial classifier could predict the subject's responses in 63% of the trials, which was close to significant (p = 0.056).
